# Secondhand smoke exposure among never-smoking adolescents in Wuhan, China

**DOI:** 10.1038/s41598-022-18612-y

**Published:** 2022-08-20

**Authors:** Xin Mei, Gong Chen, Qing Zhong, Yi-Lin Li, Jun-Lin Li

**Affiliations:** 1 Department of Health Education, Wuhan Center for Disease Control and Prevention, Wuhan, 430024 China; 2 Medical Department, The Central Hospital of Wuhan, Tongji Medical College, Huazhong University of Science and Technology, Wuhan, 430014 China

**Keywords:** Health care, Disease prevention, Public health

## Abstract

Without smoke-free legislation in Wuhan, China, we investigated secondhand smoke (SHS) exposure at home, school, and public places for never-smoking school-going adolescents in 2019. A cross-sectional study was carried out within the Global Youth Tobacco Survey (GYTS) framework. Weighted univariate, bivariate and multivariable analyses were conducted. The prevalence of SHS exposure among never-smoking adolescents at home, school and public places was 25.7%, 31.9% and 48.9%, respectively. Multivariable logistic regression analysis showed that parents smoking, peers smoking and observing teachers smoking in school were all significantly related to a higher probability of SHS exposure at home, school, and public places. Never-smoking adolescents who had smoking parents had 14 times (adjusted odds ratio [aOR], 14.00; 95% confidence interval [CI], 11.37–17.24) higher odds of SHS exposure at home; Never-smoking adolescents who observed teachers smoking in school had about 10 (aOR = 9.76; 95% CI = 7.13–13.36) and four times (aOR = 3.55; 95% CI = 2.77–4.55) higher odds of SHS exposure in school and public places, respectively. Adopting comprehensive smoke-free legislation in public places and smoke-free home rules and implementing and supervising smoke-free school policies may further reduce SHS exposure among adolescents.

## Introduction

Smoking is a leading cause of premature mortality and morbidity, and despite clear international evidence of the harms of environmental tobacco smoke, smoking numbers worldwide remain high^1,2^. In 2019, 1.4 billion adults aged 15 years and above and 24 million adolescents aged 13–15 were tobacco users worldwide^2^, and more than 300 million tobacco users lived in China^1^. One million people die from tobacco-related diseases yearly, and 100,000 never-smokers die from secondhand smoke (SHS) exposure in China^2^. SHS, also known as environmental tobacco smoke, is a mixture of the smoke from the burning end of a cigarette, cigar, or pipe and the smoke breathed out by the smoker^3^. Exposure to SHS can cause severe and fatal diseases in adults, including respiratory disease, cardiovascular disease, and cancer, which will further significantly increase morbidity and mortality^4^.

Exposure to SHS among adolescents is also an important issue. About 63% of adolescents worldwide were exposed to SHS, and 603,000 deaths were attributed to SHS, of which 28% occurred in children younger than 15^5,6^. Disability-adjusted life-years lost because of exposure to SHS amounted to 10.9 million worldwide, of which 61% were children younger than 15^5^. Adolescents are particularly vulnerable to SHS and are at increased risk for depression, mental illness, insufficient sleep, suicide attempts, respiratory symptoms and infections, and obesity^7–11^. It also creates an environment in which smoking is socially acceptable, thereby increasing the likelihood that adolescents will start smoking^12^. As adolescents are psychologically and biologically the most vulnerable to secondhand smoke exposure^6^, it is crucial to protect them from the adverse effects of SHS during the critical developmental stage and the subsequent burden of the disease continued into adulthood by adopting and implementing smoke-free policies.

The World Health Organization (WHO) issued the Framework Convention on Tobacco Control (FCTC) to curb the harm of tobacco and SHS. It showed how to protect young people from low-priced tobacco, tobacco advertising, promotion, and sponsorship (TAPS), and SHS exposure. The Global Youth Tobacco Survey (GYTS) is a standardized school-based survey jointly developed by the WHO and the U.S. Centers for Disease Control and Prevention (CDC) to provide the data needed to support the design, implementation, and evaluation of tobacco prevention and control programs (TCPs)^6^. The GYTS has been widely implemented in most WHO member states^13^. The results of the GYTS showed that environmental tobacco smoke exposure worldwide was very high^14^. The global prevalence of SHS exposure among school-going adolescents was 44.1% and 54.2% at home and in public places, respectively^15^. The data among never-smoking adolescents were 39.1% and 49.5%, respectively^16^. Parents' or peers' tobacco use, knowledge of the harms of SHS, and positive attitudes toward the smoking ban were related to higher exposure to SHS^16^.

The National Youth Tobacco Survey in China (NYTS) uses the standard sampling methods, questionnaires and data analysis methods of the GYTS^17^, which showed suitability by the reliability and validity analysis^18^. The NYTS was first conducted in the fall of 2013 and again in the fall of 2019, providing representative data of all junior high school students in the 31 provinces of China. However, there are few articles about China based on the NYTS between 2000 and 2020, and the topic was about smoking prevalence, electronic cigarettes, smoking experimentation, and comparison of tobacco use^17,19–21^. There is limited data on exposure to SHS among adolescents in China, and there is no data in Wuhan either. Wuhan is the capital of Hubei and the core city of central China, with more than 12 million permanent residents and 200,000 junior high school students^22^. In conjunction with the NYTS in 2019, the Wuhan Youth Tobacco Survey (YTS) was developed to provide the data necessary to estimate adolescents' tobacco use comprehensively. This study aims to provide representative baseline data on SHS exposure among never-smoking adolescents in Wuhan to guide subsequent research and tobacco control efforts. To our knowledge, it was the first city-level investigation into YTS in Wuhan.

## Methods

### Sampling design

The Wuhan YTS was a cross-sectional, school-based survey of junior high school students using the GYTS standardized protocol and questionnaire. The universe for the survey consisted of junior high school students in grades 7–9 in all public and private schools in 15 districts of Wuhan. The universe of this paper was junior high school students who had never smoked. The sampling frame consisted of primary sampling units (PSUs) made up of schools that can supply a whole complement of students in grades 7–9. Schools with fewer than 40 students were not included in the sampling frame. A representative sample was generated using a two-stage cluster sample design. In the first stage, 45 PSUs (overall 277 PSUs) were selected from 15 administrative districts using the probability proportional to size (PPS) method. In the second stage, one class was selected from each grade in PSU with the simple random sampling method, and a total of 135 classes were selected. All students in the selected class were invited to participate in the survey. The sampling frame, obtained from Wuhan Municipal Education Bureau, contained all school information, including the number of students, school type and other contact information.

### Survey instrument

The 2019 NYTS survey instrument included 45 questions. The first four questions on the survey collected adolescents' demographic characteristics, and the rest measured a comprehensive set of tobacco-related topics. Specific topics included: tobacco product use, cessation attempts, knowledge of and attitudes toward tobacco use, SHS exposure, harm perceptions, and exposure to pro- and anti-tobacco advertising and media^13^. There are all single-choice questions and no skip mode. The questionnaire was prepared in Chinese, China's common and official language. The self-administered questionnaire was conducted using traditional paper-and-pencil (PAPI). One class period (about 40 min) was given to students to complete the questionnaire. Students absent on the survey day were not asked to complete the questionnaire after returning to school.

### Survey administration

The research manual of GYTS^23^, which includes detailed management procedures, was followed strictly. The data collector and investigation coordinator training was conducted on September 12– 14, 2019, to ensure the survey protocol and procedures would be identical. Survey administration in the schools began on September 26, 2019, immediately after the training, and continued until November 21, 2019. Students participated in the survey anonymously and voluntarily. The survey did not collect personal information, such as name, ID number or other identifying information, and school staff were not present during the survey. Informed consent was obtained from all participants and parents or legal guardians of participants younger than 16. The study was conducted following the Declaration of Helsinki, and the processes, security elements, and sampling design were reviewed and approved by Wuhan CDC Institutional Review Board (IRB).

### Variables

As school-going adolescents spend much time at home and school^24–26^, they also inevitably go to public places. The dependent variables were SHS exposure at home, school and public places^6,27,28^. Similar to earlier studies^6,26,29–31^, the following independent variables potentially related to the dependent variables were included. These variables were gender, grade, knowledge about the harm of SHS, parent or peer smoking behaviour, whether the adolescents observed teachers smoking in school, school type, disposable income, exposure to anti-smoking media and tobacco advertising and promotion. The detailed coding of the survey items is shown in Table 1.

**Table 1:** Question-wording, response options and coding.

Variables	Survey items	Original response options	Dichotomized measure
**Item used to obtain never-smoking adolescents**			
Smoking status	Have you ever tried or experimented with cigarette smoking, even one or two puffs?	No; Yes	No = never smoker;Yes = ever smoker
**Dependent variable**			
Exposure to SHS at home	During the past seven days, how many days did someone smoke tobacco products in your home while you were there?	0 day; 1–2 days; 3–5 days; 6–9 days; 7 days	No = 0 day; Yes = ≥ 1 days
Exposure to SHS in public places	During the past seven days, how many days did you smell the smoke from someone smoking tobacco products in an indoor or outdoor public place? During the past seven days, how many days did you ride in a vehicle when someone was smoking a tobacco product?	0 day; 1–2 days; 3–5 days; 6–9 days; 7 days	No = 0 days for both items; Yes = ≥ 1 days for either item
Exposure to SHS in school	During the past 30 days, have you noticed anyone smoking inside or outside the school building?	No; Yes	No = No; Yes = Yes
**Independent variables**			
Knowledge about the harm of SHS	Do you think the smoke from other people's cigarettes harms you?	Definitely not; Probably not; Probably yes; Definitely yes	No = definitely not; Yes = any other responses
Peer smoking	Do any of your closest friends smoke cigarettes?	None of them; Some of them; Most of them; All of them	No = none of them; Yes = any other response
Parental smoking	Do your parents smoke?	None; Both; Father only; Mother only; I don't know*	No = None or I don't know; Yes = Both or Father only or Mother only; . = I don't know
Exposed to antismoking media messages	During the past 30 days, have you seen anti-smoking media messages (e.g., television, radio, Internet, billboards, poster, etc.)?	No; Yes	No = No; Yes = Yes
Observing teachers smoking in school	How often do you usually see teachers smoking inside or outside the school buildings during school hours?	Almost every day; Sometimes; Never; I do not know*	No = Never or I do not know; Yes = Almost every day or sometimes; . = I don't know
Disposable income (CNY)	Within a week, on average, how much disposable income do you have?	None; Less than 10; 11 to 20; 21 to 30; 31 to 40; 41 to 50; More than 50	None; Less than 20; 21 to 50; More than 50;
Exposed to tobacco advertising and promotion	During the past 30 days, when you go to tobacco retail outlets (such as kiosks or supermarkets), have you seen ads or promotions for cigarettes or other tobacco products? During the past 30 days, when you are using the Internet, have you seen ads or videos for cigarettes or other tobacco products?	No; Yes	No = no for both items; Yes = any other response

“No” responses were coded as “0” and “Yes” responses as “1” for the multivariable logistic regression model.

*SHS* secondhand smoke, *CNY* Chinese Yuan, 1 United States dollar = 6.7 Chinese yuan.

*Set “I don't know” as missing values.

### Response rates

The survey's response rate was explained at the school and student levels. At the school level, 44 of the 45 schools selected (97.8%) participated in the survey. The remaining one school was considered to refuse because of losing instructional time. At the student level, 6,126 eligible students were invited to participate in the survey, of which 6,069 did so, and the response rate of students was 99.1%. The remaining 57 students did not participate because they were out of school on the survey day or unwilling to participate. When the response rates of students and schools were combined, the overall response rate was 96.9%, thus considered sufficient for weighting purposes.

### Data analysis

The original sample consisted of 6,069 school-going adolescents. We restricted our analysis to never-smoking adolescents, comprising 5,632 individuals (92.8% of the original sample). Data cleansing rules were created to ensure accuracy and eliminate internal inconsistencies. The sampling weight was considered since the survey adopted a multistage cluster sampling procedure. The data were weighted to represent the population of junior high school students in Wuhan by a three-step process: (1) calculation of the base weight (school and class levels), (2) nonresponse adjustment (school and student levels), and (3) post-stratification calibration adjustment of sample totals to the known population totals^15^. Rao-Scott chi-square tests were used for differences in characteristics among subgroups in bivariate analysis. Multivariable logistic regression analysis was conducted to identify the factors related to SHS exposure. Statistical inferences were assessed by a two-sided 5% significance level. Data analyses were conducted with SAS software version 9.4 (SAS Institute Inc. Cary, NC).

## Results

### Sample characteristics

A total of 5,632 never-smoking adolescents aged about 12–15 (99.2% were in the 12–15 age group) were included in the study, representing 205,552 never-smoking adolescents in Wuhan. About half of adolescents (53.3%) who had never smoked were male, and students in grades 7, 8 and 9 each accounted for about one-third. More than two-fifths (41.7%) of adolescents who had never smoked had smoking parents, and about one-ninth (10.6%) had smoking peers. Moreover, 97.6% had knowledge about the harm of SHS exposure, and 29.6% observed teachers smoking in school (Table 2).

**Table 2:** Demographic characteristics of secondhand smoke (SHS) exposure among never-smoking adolescents, in Wuhan, China.

Characteristics	Frequencies		Weighted percent (%)*
	Unweighted (n)	Weighted (N)	
**Overall**	5,632	205,552	100.0
**Gender**			
Female	2,675	95,955	46.7
Male	2,957	109,597	53.3
**Grade**			
9	1,790	63,733	31.0
8	1,891	68,415	33.3
7	1,951	73,404	35.7
**Knowledge about the harm of SHS**			
No	130	5,016	2.4
Yes	5,502	200,536	97.6
**Peer smoking**			
No	4,989	183,829	89.4
Yes	643	21,723	10.6
**Parent smoking**			
No	3,083	116,577	56.7
Yes	2,452	85,807	41.7
**Exposed to antismoking media messages**			
No	1,378	48,032	23.4
Yes	4,254	157,520	76.6
**Observing teachers smoking in school**			
No	3,199	122,744	59.7
Yes	1,817	60,944	29.6
**School type**			
Public school	4,505	172,169	83.8
Private school	1,127	33,383	16.2
**Disposable income (CNY)**			
0	1,061	38,011	18.5
1–20	2,186	82,379	40.1
21–50	1,495	53,575	26.1
More than 50	890	31,587	15.4
**Exposed to tobacco advertising and promotion**			
No	3,188	112,532	54.7
Yes	1,111	39,874	19.4

*CNY* Chinese Yuan, 1 United States dollar = 6.7 Chinese yuan.

*The percentages don't add to 100 due to missing value.

### Bivariate analysis

Overall, 25.7%, 31.9% and 48.9% of the school-going adolescents who had never smoked were exposed to SHS at home, school and public places, respectively. Table 3 presents the bivariate analysis of potential factors related to SHS exposure. Of the ten variables considered, all but three (gender, grade, and school type), one (grade), and two (gender and grade) were statistically significantly related to SHS exposure at home, school, and public places, respectively (*P* < 0.05).

**Table 3:** Prevalence estimates of secondhand smoke (SHS) exposure among never-smoking adolescents (Weighted).

Characteristics	SHS exposure at home		SHS exposure in school		SHS exposure in public places	
	n (%)*	*P*	n (%)*	*P*	n (%)*	*P*
**Overall**	1,544 (25.7)	–	1,928 (31.9)	–	2,927 (48.9)	–
**Gender**						
Female	743 (26.1)	0.507	853 (29.8)	0.004	1,412 (49.5)	0.460
Male	801 (25.3)		1,075 (33.7)		1,515 (48.4)	
**Grade**						
9	453 (24.4)	0.151	690 (34.8)	0.120	938 (49.2)	0.425
8	577 (28.2)		696 (33.7)		1,035 (51.1)	
7	514 (24.6)		542 (27.6)		954 (46.6)	
**Knowledge about the harm of SHS**						
No	21 (14.7)	0.007	62 (49.0)	< 0.001	43 (30.1)	< 0.001
Yes	1,523 (26.0)		1,866 (31.4)		2,884 (49.4)	
**Peer smoking**						
No	1,282 (24.1)	< 0.001	1,488 (27.8)	< 0.001	2,444 (46.0)	< 0.001
Yes	262 (39.7)		440 (66.4)		483 (74.1)	
**Parent smoking**						
No	200 (6.0)	< 0.001	879 (25.7)	< 0.001	1,291 (38.6)	< 0.001
Yes	1,319 (52.5)		1,024 (40.4)		1,592 (63.1)	
**Exposed to antismoking media messages**						
No	414 (28.7)	0.034	552 (38.1)	< 0.001	778 (54.2)	0.001
Yes	1,130 (24.8)		1,376 (29.9)		2,149 (47.3)	
**Observing teachers smoking in school**						
No	680 (19.8)	< 0.001	487 (14.7)	< 0.001	1,228 (35.8)	< 0.001
Yes	685 (37.1)		1,253 (67.3)		1,345 (72.8)	
**School type**						
Public school	1,186 (24.8)	0.054	1,450 (30.0)	0.039	2,188 (45.9)	< 0.001
Private school	358 (30.2)		478 (41.5)		739 (64.6)	
**Disposable income (CNY)**						
0	240 (20.3)	< 0.001	371 (31.9)	< 0.001	510 (44.8)	< 0.001
1–20	553 (23.3)		637 (26.8)		1,006 (42.4)	
21–50	440 (27.8)		508 (32.4)		820 (52.5)	
More than 50	311 (35.1)		412 (43.9)		591 (65.0)	
**Exposed to tobacco advertising and promotion**						
No	879 (26.5)	< 0.001	1,026 (29.9)	< 0.001	1,652 (49.5)	< 0.001
Yes	399 (34.0)		538 (45.9)		754 (64.8)	

*SHS* secondhand smoke, *CNY* Chinese Yuan, 1 United States dollar = 6.7 Chinese yuan.

*Unweighted frequencies, weighted percentages.

### Multivariable logistic regression analysis

While gender, grade, and school type were not significantly related to SHS exposure, these variables were retained in multivariable logistic regression models for possible confounding^13^. The multivariable logistic regression analysis showing how individuals' characteristics were related to SHS at home, school, and public places, respectively, among never-smoking adolescents, was shown in Table 4. The likelihood-ratio tests of the logistic regression were performed, and the results showed that all *P* < 0.001. It can be considered that the logistic regression equations were statistically significant.

**Table 4:** Risk factors of secondhand smoke (SHS) exposure among never-smoking adolescents (Weighted).

Characteristic	SHS exposure at home		SHS exposure in school		SHS exposure in public places	
	*OR* (95% *CI*)	*P*	OR (95% CI)	P	*OR* (95% *CI*)	*P*
**Gender**						
Female	1 (ref)	–	1 (ref)	–	1 (ref)	–
Male	0.93 (0.74–1.17)	0.542	1.15 (0.97–1.38)	0.116	0.92 (0.80–1.06)	0.226
**Grade**						
9	1 (ref)	–	1 (ref)	–	1 (ref)	–
8	1.39 (1.14–1.70)	0.001	1.14 (0.82–1.58)	0.452	1.24 (0.94–1.65)	0.135
7	1.47 (1.10–1.96)	0.009	1.23 (0.93–1.64)	0.146	1.37 (0.95–1.96)	0.088
**Knowledge about the harm of SHS**						
No	1 (ref)	–	1 (ref)	–	1 (ref)	–
Yes	2.77 (1.38–5.56)	0.004	0.71 (0.35–1.45)	0.350	3.32 (1.61–6.85)	0.001
**Peer smoking**						
No	1 (ref)	–	1 (ref)	–	1 (ref)	–
Yes	1.47 (1.02–2.14)	0.041	2.39 (1.66–3.44)	< 0.001	1.72 (1.22–2.44)	0.002
**Parent smoking**						
No	1 (ref)	–	1 (ref)	–	1 (ref)	–
Yes	14.00 (11.37–17.24)	< 0.001	1.54 (1.14–2.07)	0.005	2.22 (1.80–2.74)	< 0.001
**Exposed to antismoking media messages**						
No	1 (ref)	–	1 (ref)	–	1 (ref)	–
Yes	0.99 (0.78–1.25)	0.919	0.81 (0.63–1.05)	0.106	0.93 (0.73–1.18)	0.562
**Observing teachers smoking in school**						
No	1 (ref)	–	1 (ref)	–	1 (ref)	–
Yes	1.66 (1.32–2.08)	< 0.001	9.76 (7.13–13.36)	< 0.001	3.55 (2.77–4.55)	< 0.001
**School type**						
Public school	1 (ref)	–	1 (ref)	–	1 (ref)	–
Private school	1.40 (1.17–1.68)	< 0.001	1.40 (1.00–1.96)	0.053	2.02 (1.29–3.15)	0.002
**Disposable income (CNY)**						
0	1 (ref)	–	1 (ref)	–	1 (ref)	–
1–20	1.08 (0.86–1.35)	0.515	1.03 (0.79–1.33)	0.848	0.97 (0.74–1.27)	0.813
21–50	1.27 (0.98–1.65)	0.076	1.18 (0.86–1.63)	0.300	1.37 (1.08–1.73)	0.009
More than 50	1.64 (1.20–2.23)	0.002	1.32 (0.95–1.84)	0.097	1.96 (1.54–2.50)	< 0.001
**Exposed to tobacco advertising and promotion**						
No	1 (ref)	–	1 (ref)	–	1 (ref)	–
Yes	1.10 (0.93–1.29)	0.268	1.51 (1.21–1.89)	< 0.001	1.53 (1.28–1.83)	< 0.001

*SHS* secondhand smoke, *CNY* Chinese Yuan, 1 United States dollar = 6.7 Chinese yuan, *OR* odds ratio, *CI* confidence interval, *ref* referent group.

When considering the risk factors for SHS at home, We found the most increased odds of SHS prevalence among never-smoking adolescents who had smoking parents (adjusted odds ratio [aOR], 14.00; 95% confidence interval [CI], 11.37–17.24), followed by those who had knowledge about the harm of SHS (aOR = 2.77; 95% CI = 1.38–5.56), observed teachers smoking in school (aOR = 1.66; 95% CI = 1.32–2.08), had more than 50 Chinese Yuan (CNY) disposable income per week (aOR = 1.64; 95% CI = 1.20–2.23), had smoking peers (aOR = 1.47; 95% CI = 1.02–2.14), attended private school (aOR = 1.40; 95% CI = 1.17–1.68), and were in grade 7 (aOR = 1.47; 95% CI = 1.10–1.96) or grade 8 (aOR = 1.39; 95% CI = 1.14–1.70).

Never-smoking adolescents, those who observed teachers smoking in school (aOR = 9.76; 95% CI = 7.13–13.36), those who had smoking peers (aOR = 2.39; 95% CI = 1.66–3.44) or smoking parents (aOR = 1.54; 95% CI = 1.14–2.07), and those who were exposed to tobacco advertising and promotion (aOR = 1.51; 95% CI = 1.21–1.89), had higher odds of SHS exposure in school.

Never-smoking adolescents who observed teachers smoking in school (aOR = 3.55; 95% CI = 2.77–4.55) were most significantly related to increased SHS exposure in public places, followed by those who had knowledge about the harm of SHS (aOR = 3.32; 95% CI = 1.61–6.85), those who had smoking parents (aOR = 2.22; 95% CI = 1.80–2.74), those who attended private school (aOR = 2.02; 95% CI = 1.29–3.15), those who had more than 50 (aOR = 1.96; 95% CI = 1.54–2.50) or 21 to 50 (aOR = 1.37; 95% CI = 1.08–1.73) CNY disposable income per week, those who had smoking peers (aOR = 1.72; 95% CI = 1.22–2.44), and those who were exposed to tobacco advertising and promotion (aOR = 1.53; 95% CI = 1.28–1.83), respectively.

## Discussion

Exposure to tobacco smoke remains a global health problem. Monitoring tobacco use is critical to combating the tobacco epidemic and assessing the implementation of FCTC and MPOWER measures^2^. Exploring the potentially related factors of local SHS exposure is significant for formulating and implementing tobacco control policies and SHS exposure intervention measures. To the best of our knowledge, this is the first study to estimate the prevalence and related factors of SHS exposure in a representative sample of never-smoking adolescents in Wuhan, China. The survey showed that SHS exposure was prevalent among never-smoking adolescents in Wuhan, China. Approximately one-fourth (25.7%), one-third (31.9%), and one-half (48.9%) of adolescents who had never smoked were exposed to SHS at home, school and public places, respectively. The NYTS showed that in China, 44.4%, 57.2% and 58.3% of adolescents who had never smoked were exposed to SHS at home, school and public places, respectively^32^. According to the latest surveys in 142 countries, the prevalence of SHS exposure at home and in public places was 33.1% and 57.6%, respectively^6^. According to previous studies, differences in the prevalence of SHS exposure can be attributed to regional differences in demographic characteristics, economic levels, lifestyles, and smoking prevalence^26,29,33^.

Previous studies have shown that SHS exposure can be a proxy for social pressures to smoke from peers, teachers, or parents^29,34^. Similar to these studies, we found in the multivariable analysis that parent or peer smoking and observing teachers' smoking in school were significantly related to adolescents' exposure to SHS. The strongest determinants related to exposure to SHS at home among adolescents who had never smoked were parent smoking, consistent with earlier studies, and the strongest determinants in school and public places were observing teachers smoking in school^16,29,33^. There may be the following reasons. First, previous studies showed that SHS exposure was positively related to local tobacco control policies^16^. Currently, there are no national-level smoke-free regulations or laws in China^35^. For public places, the Smoking Ban in Public Places of Wuhan was promulgated by Wuhan Municipal People's Government in 2005, stipulating that smoking was prohibited in some indoor public places and school teaching places^36^. However, the Smoking Ban had a low level of legal effect and a limited range of tobacco control places, the punishment measures were light, and the principal enforcement department was not transparent^35^. For households, no smoke-free regulations at home were adopted in Wuhan, and 62.2% of households had no voluntary smoke-free home rules^37^. Second, school-going adolescents usually spend much time at home and school^5,26^, but they usually lack the ability to avoid SHS as they have limited say in smoking, which may further make them tolerant of smoking and SHS. At last, the smoking behaviour of parents, peers and teachers will affect adolescents' attitudes towards smoking favourably, and adolescents would tolerate, agree, and even imitate this behaviour. They may also not deliberately or actively avoid SHS in public places.

There was evidence that reducing adult smoking can help reduce adolescents' exposure to SHS to a large extent^2,38^. The way to reduce adult smoking may be to adopt further and widely implement the WHO FCTC and its guidelines, such as raising tobacco taxes, adopting comprehensive smoke-free legislation in public places, completely banning TAPS, and providing effective programs to assist smokers to quit^2,39^. However, the smoke-free regulations or laws cannot be extended to private residences and social life^29^. Therefore, abstaining from smoking at home mainly relied on non-regulatory measures. Studies have shown that public education campaigns, community intervention, social norms, and smoke-free home rules can substantially reduce SHS exposure at home^4,6,29,40^. Corresponding strategies, such as educating parents about the risks of smoking, SHS exposure and the benefits of smoke-free home rules, should be implemented to motivate them to ban smoking at home voluntarily^30^. As for teachers' smoking in schools, the Ministry of Education of the people's Republic of China posted a notice on the smoke-free school in 2014^41^, and the effect seemed unsatisfactory. Relevant government departments need to strengthen the implementation and supervision of the smoke-free policy and strengthen training, publicity and education to achieve a truly 100% smoke-free school. Furthermore, great efforts should be made to monitor and evaluate the effects of the above measures to sustain broad public support and formulate best practices^42^.

In the multivariable analysis, the negative relationship between SHS exposure (especially at home and school) and adolescents' knowledge about the harm of SHS was consistent with the results reported by Veeranki SP et al.^16^ and Mamudu HM et al^4^. The universe of the survey was adolescents, who were vulnerable groups and had limited ability to avoid smoking environment, which meant that knowledge about the harm of SHS has a limited impact on behaviour^16^. We also found that exposure to SHS in school and public places were significantly higher for adolescents exposed to tobacco advertising and promotion, consistent with earlier studies^43,44^. Exposure to tobacco advertising and promotion may shift adolescents' attitudes toward smoking favourably by shaping adolescents' curiosity about smoking, perceived norms, and perceptions about the benefits of smoking, thus making adolescents accept and tolerate smoking and SHS^45,46^. According to WHO FCTC, there should be a broad ban on all tobacco advertising, promotion, and sponsorship^2^. In response to the WHO FCTC, the Advertising Law of the People's Republic of China was revised in September 2015, in which Article 22 stipulates that tobacco advertisements in the mass media, public places, public transport, and outdoors are entirely prohibited, and any form of tobacco advertisements to adolescents is prohibited. However, a study conducted by the Chinese Association on Tobacco Control reported that tobacco advertisements were found in up to 64.9% of tobacco retail outlets in China, and up to 57.6% of tobacco retail outlets did not have any signs prohibiting the sale of cigarettes to adolescents^47^. The WHO FCTC should be strictly implemented, the enforcement of the Advertising Law should be strengthened, and the supervision and enforcement efforts should be strengthened. In addition, the results indicated that adolescents exposed to anti-smoking media messages were not significantly related to SHS exposure in the study. Based on the three findings above, parents' smoking behaviour, adolescents' exposure to tobacco advertising and promotion and lack of ability to avoid SHS may not only make them tolerant of SHS exposure but also may hinder the effectiveness of efforts to reduce SHS exposure by anti-smoking media messages and knowledge about the harm of SHS^4^.

At last, the multivariable analysis also indicated that adolescents' school type and disposable income might affect SHS exposure. Adolescents' higher disposable income was related to a higher risk of SHS exposure. One explanation may be that adolescents with a higher disposable income had the purchasing ability to eat in restaurants, shop in stores, watch movies in cinemas and play in entertainment venues (such as video arcades, internet bars, and children's parks), which made them more likely to be exposed to SHS in these public places. As adolescents' disposable income may not accurately reflect their family's socioeconomic status^48^, another reasonable explanation was that higher disposable income might indicate less parental engagement and poorer parental supervision^49^, allowing themselves to smoke at home. Compared with adolescents attending public schools, adolescents in private schools were related to a higher risk of exposure to SHS. The poorly implemented and supervised tobacco control policies in private schools and students' continued and passive tolerance of SHS may lead to the phenomenon^6,49^.

This study is subject to several limitations. First, the data were from a cross-sectional survey, and the results should be interpreted cautiously. Second, adolescents' exposure to SHS was assessed based on self-reporting and may be affected by social expectations and reporting biases. Third, there was a lack of information on specific places where adolescents were exposed to SHS (except for school), indicating the need to collect specific information to support local legislation. At last, SHS exposure was assessed using a self-report questionnaire without any objective measurement, such as the levels of nicotine in the air and biomarkers (cotinine and 4- (methylnitrosamino) -1 -( 3- pyridyl) -1 -butanol) in the urine. Adolescents' exposure to SHS may be underestimated, and future studies should adjust them to provide precise estimates of SHS exposure.

## Conclusions

With few studies on SHS exposure in China, we studied the prevalence and potential factors related to SHS exposure among never-smoking school-going adolescents in Wuhan, China. The prevalence of SHS exposure among adolescents at home, school and public places was 25.7%, 31.9% and 48.9%, respectively. We substantiated that parent smoking and observing teachers smoking in school were all significantly related to adolescents' exposure to SHS at home, school and in public places. Our findings emphasize the need to strengthen tobacco control policy and program in Wuhan with a particular focus on adolescents, as they are vulnerable. It is crucial to adopt comprehensive smoke-free legislation in public places and smoke-free home rules, implement and monitor smoke-free school policy, educate the public about the risk of smoking and SHS exposure, and empower adolescents to insist on their right to smoke-free environments.

## Data Availability

The data used and/or analyzed in the current study are not publicly available because restrictions apply to the availability of these data. Data are, however, available from the corresponding author on reasonable request and with permission of the Wuhan CDC.
